# Endotracheal Excellence: Standardizing Documentation to Drive Quality in Airway Rescue Procedures Across the Health System

**DOI:** 10.7759/cureus.112953

**Published:** 2026-07-19

**Authors:** Emily Greenwald, Tim Crittenden, Palen Mallory, Michelle DeRusso, Aaron Adams, Keyaria Gray, Lakshmi Katakam, Janet Lee, Sarah Gonzalez-Bankich, Claire Washabaugh, Brad Taicher, Emily Sterrett

**Affiliations:** 1 Pediatric Emergency Medicine, Duke University Hospital, Durham, USA; 2 Clinical Informatics, Duke Health Technology Solutions, Duke Medical Center, Durham, USA; 3 Pediatric Critical Care Medicine, Duke University Hospital, Durham, USA; 4 Neonatology, Duke University Hospital, Durham, USA; 5 Head and Neck Surgery/Communication Sciences, Duke University Hospital, Durham, USA; 6 Medicine, Duke University School of Medicine, Durham, USA; 7 Pediatric Anesthesiology, Duke University Hospital, Durham, USA

**Keywords:** advanced airway management, documentation templates, electronic health record (ehr), procedure documentation, endotracheal intubation

## Abstract

Background

Out-of-operating-room airway management is a time-sensitive, life-saving procedure performed in many clinical units in acute care hospitals. Local airway management practice should be understood and monitored to identify opportunities for quality improvement.

Objectives

As a first step to drive excellence in advanced airway practice across our three-hospital health system, we sought to establish documentation standards for emergency airway procedures.

Methods

Representatives in advanced airway practice (emergency medicine, anesthesia, critical care, otolaryngology, respiratory therapy) and clinical informatics investigated current institutional airway procedure documentation and national best practices. We developed a high-reliability note template based on the electronic health record (EHR) to document the details of an airway event and support participation in national emergency airway registries. It was designed and implemented using *in situ* simulation with high-utilizer groups. Simulation and one-on-one end-user feedback gathered through interviews and surveys informed subsequent iterations. The new note template capitalized on existing emergency department (ED) workflows, so it was launched first in EDs across the health system. Using a similar implementation plan and through collaboration with relevant stakeholders, the note was adapted for unit-specific performance.

Results

The note is used as standard practice in EDs, delivery rooms, neonatal and pediatric intensive care units (ICUs), and is available for all hospital-based providers in the health system. The note has been used in over 3,000 patient encounters. Note data is available on demand, an important resource that drives both continuous performance improvement efforts and efficiency for national registry participation. The information contained within the note drives various quality improvement and safety initiatives that reach patients.

Conclusion

Standardizing documentation for airway procedures using shared electronic data elements in the EHR is important as it provides a discrete data stream for capturing our current practice, performance outcomes, opportunities, and vulnerabilities.

## Introduction

Advanced airway management techniques, including tracheal intubation (TI), supraglottic airway device placement, laryngoscopy, bronchoscopy, and surgical airway procedures, are mostly performed in controlled operative settings. However, outside the operating room (OR), airway management is a life-saving intervention for critically ill patients. In non-operative settings, such as intensive care units (ICUs) and emergency departments (EDs), airway procedures are associated with greater variability in personnel, equipment availability, and environmental conditions, contributing to increased procedural risk [1].

Since the advent of the “problem-oriented” medical record in the 1960s and the widespread adoption of electronic health records (EHRs) in the 1970s and 1980s, information in the patient chart has increased in quantity [2]. However, challenges persist, including information completeness, consistency, accuracy, and accessibility [3]. To improve documentation of airway procedures specifically, individual institutions, national registries, and professional associations have implemented varied approaches, including development of standard elements to be included in documentation, comparison of written documentation and video review, and introduction of alerts for difficult airways [4-14]. Many of the largest multi-centered airway registries driving quality improvement of out-of-OR airway events still rely on paper-based post-intubation documentation to collect detailed data around airway events [15-18].

Historically in our health system, EHR airway documentation practices were unit-specific, relying heavily on user-created templates and free-text entry. This led to an inability to track, communicate, and learn from airway events. Standardizing terminology and documentation with a tool that integrates electronic data elements is important because it provides a data stream for capturing current practice, performance outcomes, opportunities, and vulnerabilities. The institutional airway task force aimed to establish an ideal state of airway management documentation for out-of-OR events with retrievable and reportable data elements for quality benchmarking and improvement. This study was reported in accordance with the SQUIRE 2.0 guidelines [19].

## Materials and methods

Our health system consists of three hospitals: one large 1000-bed quaternary academic hospital and trauma center, including an embedded 200-bed academic quaternary children’s hospital, and two medium-sized (350- and 200-bed) community-based hospitals, of which one has labor and delivery services and a level 2 Neonatal Intensive Care Unit (NICU). All three hospitals have general EDs and adult ICUs; the quaternary hospital has pediatric ICUs and dedicated pediatric ED (PED) services. Combined, the three EDs have 147,000 arrivals per year. All three hospitals share the same EHR platform with overlapping informatics infrastructure. 

The institutional airway taskforce was created in 2021 as a pediatric-focused safety and quality effort in response to an adverse airway event in the PED. The group was established to standardize the approach to out-of-OR advanced airway management to enhance communication, ensure competencies and skills, and thereby improve critical patient outcomes. The task force is endorsed, but not directly sponsored, by the academic hospital's executive leadership. It is co-chaired by medical directors in pediatric emergency medicine and pediatric anesthesiology and includes physician representatives from pediatric and neonatal critical care, pediatric otolaryngology, as well as support from respiratory therapy and informatics/EHR specialists. Most physicians in the task force have completed project-based training in quality improvement and patient safety methodology, and some have expert-level competency in academic improvement science methods. Additionally, the airway taskforce partnered with a nurse informaticist with expertise in clinical software architecture to draft a novel out-of-OR airway procedure note.

The first step towards creating the documentation tool was to define the current and ideal state of airway documentation. Existing documentation practices in each clinical unit were crosswalked by stakeholders and airway task force members. The contents of existing notes varied with respect to consistency of documentation and amount of detail describing failed attempts, airway difficulty, patient condition before attempt, and procedural changes made between attempts. To inform the ideal state, we considered the National Emergency Airway Registries (e.g., NEAR, NEAR4Kids, NEAR4NEOS, NEAR4PEM) [15-18] for critical content and to ensure that local data collection supported participation in these registries (Table 1). We then explored informatics capabilities to optimally integrate the new note into the existing EHR system, Epic [20], with a focus on the ability to generate retrievable data elements. The ideal state also included communication of critical patient information between care teams for real-time retrieval during subsequent airway events. The ideal note would be easy to use and interpret by three key audiences: the documenter, clinician reviewers involved in the care of the patient, and reporting groups. 

**Table 1:** Airway event documentation practices for each unit prior to implementation of standardized airway note Elements (leftmost column) are based on national airway registry standards. TRUE labels represent elements that are included, while dashes represent elements that are not included. ED = emergency department, PED = pediatric emergency department, PICU = pediatric intensive care unit, PCICU = pediatric cardiac intensive care unit, NICU = neonatal intensive care unit, ICU = intensive care unit, TI = tracheal intubation, CPR = cardiopulmonary resuscitation.

	ED / PED	PICU/PCICU	NICU	ICU	NEW NOTE
						Ability for automated data retrieval	TRUE	-	-	TRUE	TRUE
Documentation of details regarding failed attempts	-	-	-	-	TRUE
Data collection aligned with national airway registry elements:					
· Number of attempts	TRUE	TRUE	TRUE	TRUE	TRUE
· Laryngoscopist	-	-	-	-	TRUE
· Airway difficulty today	TRUE	TRUE	-	TRUE	TRUE
· Airway difficulty history	-	TRUE	-	-	TRUE
· Intubation indication	TRUE	TRUE	-	TRUE	TRUE
· TI-related adverse events	-	TRUE	-	TRUE	TRUE
· Drugs used	-	TRUE	-	TRUE	TRUE
· Device used	TRUE	-	TRUE	TRUE	TRUE
· Ongoing CPR	-	TRUE	-	-	TRUE
· Patient anatomical characteristics (e.g. widest mouth opening)	TRUE	TRUE	-	TRUE	TRUE
· Bag-Mask ventilation difficulty	-	TRUE	-	-	TRUE
· Use of apneic oxygenation	-	TRUE	-	-	TRUE
· Known cyanotic heart disease	-	TRUE	-	-	TRUE

To update and consolidate existing free-text notes, we used discrete data elements mapped behind the text to enhance reportability without impacting the text-based nature of the note. We used a form-and-note combination, meaning that the discrete elements filled in by clinicians are used to generate a text-based note which appears in the EHR. The advantages of a form-and-note combination are that it provides a consistent look for both the documenter and clinician reviewer, is infinitely adaptable to meet clinical and departmental needs, and uses a foundational library of discrete data elements for reporting. The form portion of the workflow is a structured, comprehensive layout of all the elements of clinical documentation needed to convey an entire airway procedure.

A “pre-procedural” section has location, consent, preparation, and premedication. A “procedure” section has the number of attempts (defined as the number of times a laryngoscope passes the teeth, regardless of whether an endotracheal tube was passed), and, for each attempt, the clinician role, airway method, and equipment used. A “post-procedural” section has confirmation and securement methods, sedation, and adverse events. 

The note portion of the workflow matches every element available for documentation on the form with a paired text statement (Appendix). The details of a successful attempt are displayed within a bordered ‘box’ on the note, which draws the reader’s eye to this content. Details of failed attempts appear lower down. This is an example of reducing downstream clinician/interpreter burden. As notes are configured in the same format and layout, everyone in the organization knows what to expect and where to find key information.

**Figure 1:**
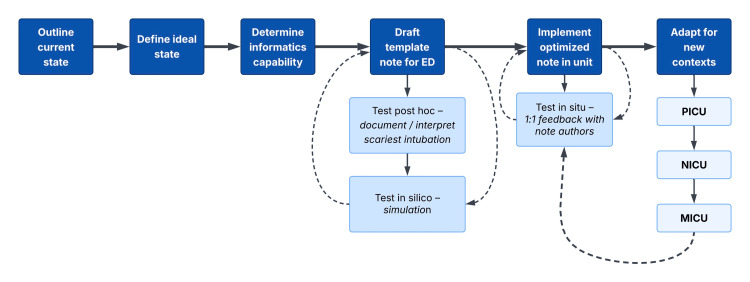
Process map for airway procedure note development, testing, and dissemination Arrows represent the flow of the process. Curved arrows represent processes that were iterative. ED = emergency department, PICU = pediatric intensive care unit, NICU = neonatal intensive care unit, MICU = medical intensive care unit. This figure is original work by the authors, created in Lucidchart (www.lucidchart.com).

Drafts of the note were tested in* post hoc*,* in silico,* and *in situ* contexts for iterative learning and optimization (Figure 1). We conducted trial sessions with resident physicians to ensure that cognitive flow of documentation matched the flow of the work and thinking at the bedside. In the first of these trial sessions (*post hoc*), a task force member asked emergency medicine residents to tell them about their “scariest intubation.” Residents used the new note in the EHR ‘playground’ context and gave feedback on their experience documenting a complex airway event in this manner. A second group of residents then reviewed the documentation and described their understanding of the event to the airway expert. The airway expert used this information to identify gaps in ability to record and report, and the drafted note template was edited accordingly. In the next iteration of trial sessions (*in silico*), residents participated in airway-based scenarios in a high-fidelity simulation lab. At the completion of a complex difficult airway simulation, the residents documented the simulated procedure as they would in a real clinical setting, and the review process was repeated. 

The note first went “live” in our health system’s three EDs. Prior to implementation, we held an orientation with department medical directors and solicited their feedback. We then developed a training video with a walk-through of the note. The previous airway procedure note was replaced with the new one in the EHR to eliminate use of multiple templates. To drive early improvement (*in situ*), we solicited one-on-one provider feedback for the first 100 completed airway notes. All communication about minor changes to the note included calls for feedback. Every critical airway event (e.g., TI with three or more attempts) was reviewed by an airway expert, and the intubating provider was contacted to ensure that the note was accurately recording these events. 

Prior to launching the new template in the pediatric and neonatal ICUs, a multidisciplinary team including attendings, fellows, and advanced practice providers worked with the airway task force to ensure applicability and adaptability of the template to these settings. Both settings had previously used locally conceived and shared templates to document airway events. In the pediatric ICU (PICU) and pediatric cardiac ICU (PCICU), instructions for the new note were distributed via email with “help sheets” attached. Training was held with intubating staff, which included airway documentation in mock patient charts. After transition to the new note, daily audits of records were performed to ensure 100% completion. If an incorrect note template was used, the proceduralist was asked to re-document using the new template. Unit-wide feedback was provided to all proceduralists if documentation error trends were noted. 

In the NICU, staff involved in note development served as trainers for their respective counterparts. The fellows and advanced practice providers were shown the new note and given a tutorial during meetings and orientations. Taskforce leaders employed four key measures to study the intervention: (1) acceptability and user-friendliness, (2) fidelity of use, (3) spread of the intervention over time, (4) ability to collect and report on airway performance. To assess acceptability and user-friendliness, we collected qualitative data through direct user feedback. This consisted of one-on-one interviews with end-users at all stages of testing and implementation. To ensure fidelity of use, the ED note was linked to procedure billing. For PED and PICU/PCICU notes, we ensured that 100% of notes were entered into national registry data. To measure spread, we tracked the number of notes completed per unit over time. Lastly, we created monthly reports detailing each airway event and ensured that the data collected could reliably be used as measures of airway performance.

## Results

During the first year of implementation, several updates were made to improve user experience and data collection. Major updates included adding new fields specific to different inpatient contexts, adding department-based scripting to show/hide different elements, forcing documentation of note time to drive improved timestamps when completion of the note is delayed, and adding tooltip hovers showing the data registry definition of certain terms to improve interpretation. Further edits were requested as national registries refined or added to their data collection practices. 

**Figure 2:**
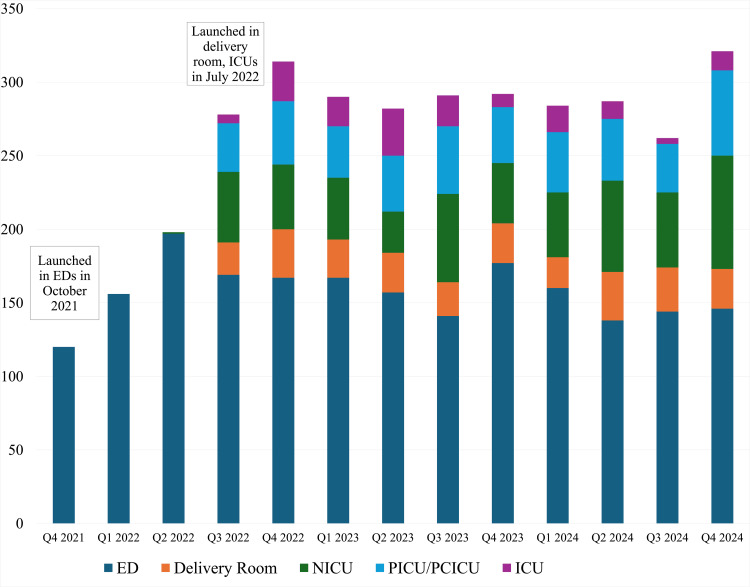
Number of airway procedure notes completed each quarter by department Each bar represents three months between October 2021 and December 2024. The left axis represents the number of notes completed. Colors represent the clinical departments. Q1 = quarter 1, Q2 = quarter 2, Q3 = quarter 3, Q4 = quarter 4.

From October 2021 to January 2025, over 3,500 airway events were captured using the note. It was launched in the ED in October 2021 (Figure 2). Dissemination to the PICU/PCICU, NICU, and delivery room occurred in July 2022. The steady state was reached in September 2022. Once steady state was reached, the average number of recorded airway events across the health system was about 100 per month, with the largest proportion in the EDs and NICU. A portion of intubations in adult ICUs are performed by anesthesiologists who document their procedures differently.

In the ED environment, the new note was electronically substituted into existing documentation workflows, creating a high-reliability adoption strategy. One-on-one follow-up between note authors and airway taskforce members occurred to screen for end-user challenges and troubleshooting. Similarly, a manual review process for all PICU intubations existed during note implementation. This manual review continued for several months to ensure fidelity and accuracy of documentation. Capitalizing on existing workflows was a key strategy to ensuring reliability in note completion.

In the NICU environment, there was a lag in adaptation of the new template due to a long-standing unit-specific template. Multiple reminders were given to staff during weekly meetings to ensure all airway events were documented consistently using the standardized airway note. In the current state, there is improved compliance with the new note. Data obtained from these notes are being used in several unit-specific quality improvement and patient safety-related projects. Reviewing this data has given insight into patient characteristics such as the type of patients being intubated in the NICU (e.g., gestational age, weight, and age during the procedure) and how these characteristics are changing over time. It has also given us information regarding the environment in which intubations take place as a neonate patient can be intubated in multiple locations such as delivery room, NICU, catheterization lab, ED etc. Furthermore, we can see what proportion of TIs receive premedication per unit guidelines. 

Within a few months of implementation, use of the new note was well engrained into the PICU/PCICU practice, with sporadic reminders required. Daily chart audits remained ongoing to ensure 100% compliance with new documentation. Airway procedure notes are collected and analyzed at routine intervals for NEAR4KIDS data monitoring/entry and to guide unit wide quality improvement work focused on TI safety.

The creation and dissemination of this universal airway procedure note has required regular collaborative work between the informaticist and physician leaders to adapt the form-and-note to meet ongoing needs of individual units. Examples include adding procedure sequencing elements for the PICU/PCICU, updating unit name for NICU, and adding a new checklist element for the PED. Along with this, behind-the-scenes software architecture work was done to take many of the local organization-based discrete data elements and petition our EHR vendor to add them to their catalog of elements. This, to facilitate the growing interest in cross-organization collaboration by using the same form-and-note concept with aligned discrete elements.

## Discussion

Our goal in establishing an ideal state of airway procedure documentation within our health system was to create a data stream to be used for quality benchmarking and improvement. Key findings are as follows: (1) retrievable data elements can be used for local learning and sending to national repositories, (2) our template allows for clinical expediency and reduced cognitive load during critical events, (3) use of a standardized template across our health system allows for accurate and easy communication of critical airway features and strategy between environments, (4) creation of an health system airway database offers opportunity for further quality improvement research. 

The note is built entirely from retrievable EHR-based data elements, enabling robust reporting on all recorded non-modifiable and modifiable aspects of the airway event. These aspects, encompassing environmental, patient-related, and procedural factors, are known to influence airway outcomes. For example, we can generate reports analyzing airway events in specific patient groups by querying almost any patient characteristic available in the EHR (e.g., neonates under 28 weeks' gestational age, or those with known difficult airways). Additionally, we can assess procedural characteristics such as laryngoscopist characteristics (specialty, trainee level), medication selection, blade choice, and use of apneic oxygenation. Finally, the system allows evaluation of first-attempt success rates, multiple-attempt rates, procedural adjustments between attempts, and trends in TI adverse events. This information can then be filtered by unit, patient category, or any relevant combination of data elements based on emerging trends from this robust and adaptable reporting system. Certain data elements considered essential can be flagged as required data elements. This comprehensive data stream provides infinite analysis opportunities for both institutional learning and broader research in combination with national repositories.

To date, most reported airway documentation standards are in national databases (NEAR, NEAR4KIDS, NEAR4NEO, etc.) or within individual departments of a hospital [21]. To our knowledge, this is the first description of the development of a single, standardized airway documentation tool intended to be used across an entire health system, encompassing several contexts such as adult and pediatric EDs and ICUs. Findings from previous studies highlight the importance of clear communication regarding difficult airways, showing that interventions aimed at alerting healthcare providers to a previously difficult TI via flags in the patient chart have resulted in earlier calls for advanced support during airway events and fewer emergency surgical airways [14,22]. Our work takes into account that patients can receive care in different locations and contexts within a health system, necessitating that critical airway information be available across all those contexts. 

With regard to critical event review, many studies have debated the benefits and drawbacks of video review versus chart review of procedure notes. Video review of intubation events is commonly used in trauma bays to drive education and quality improvement [23,24]. Video review has been shown to be more effective at capturing management errors and adverse events than chart review [25,26]. However, video recording is not available in all contexts, especially outside of the ED trauma bay setting. Additionally, the capacity to record and store videos for retrospective review is cumbersome, expensive, and unrealistic for most hospitals. In contrast, a standardized and purposefully adaptable procedure note such as the one described here is available for every airway event in every context, making it a powerful quality improvement tool. Additionally, it requires no additional equipment or major ongoing maintenance costs for hospitals. 

Interpretation of airway procedure documentation can be a challenging task in any context, especially when there are differing documentation styles and standards across an institution or health system. The primary influence of our airway procedure note was standardization of documentation of details regarding airway difficulty for each patient, which has direct implications for patient care and outcomes. 

Firstly, difficult TIs are highly predictive of subsequent difficult TIs for a given patient [27], making it critical that this information is collected and disseminated to all care teams. While the designation of a “difficult” airway is crucial, this designation alone is insufficient as it does not define what made advanced airway management challenging, describe what adverse events occurred, or provide specific guidance for future airway management for that patient. Prior to the dissemination of our note, there was no way to systematically communicate optimal airway approaches and difficult-airway considerations between providers. Intubating teams were thus required to “start from square one” each time a patient was intubated, potentially causing life-threatening delays in definitive airway management. While systematic data collection around this behavior is difficult, anecdotal reports suggest that it has supported timely decision-making in complex airway scenarios.

**Figure 3:**
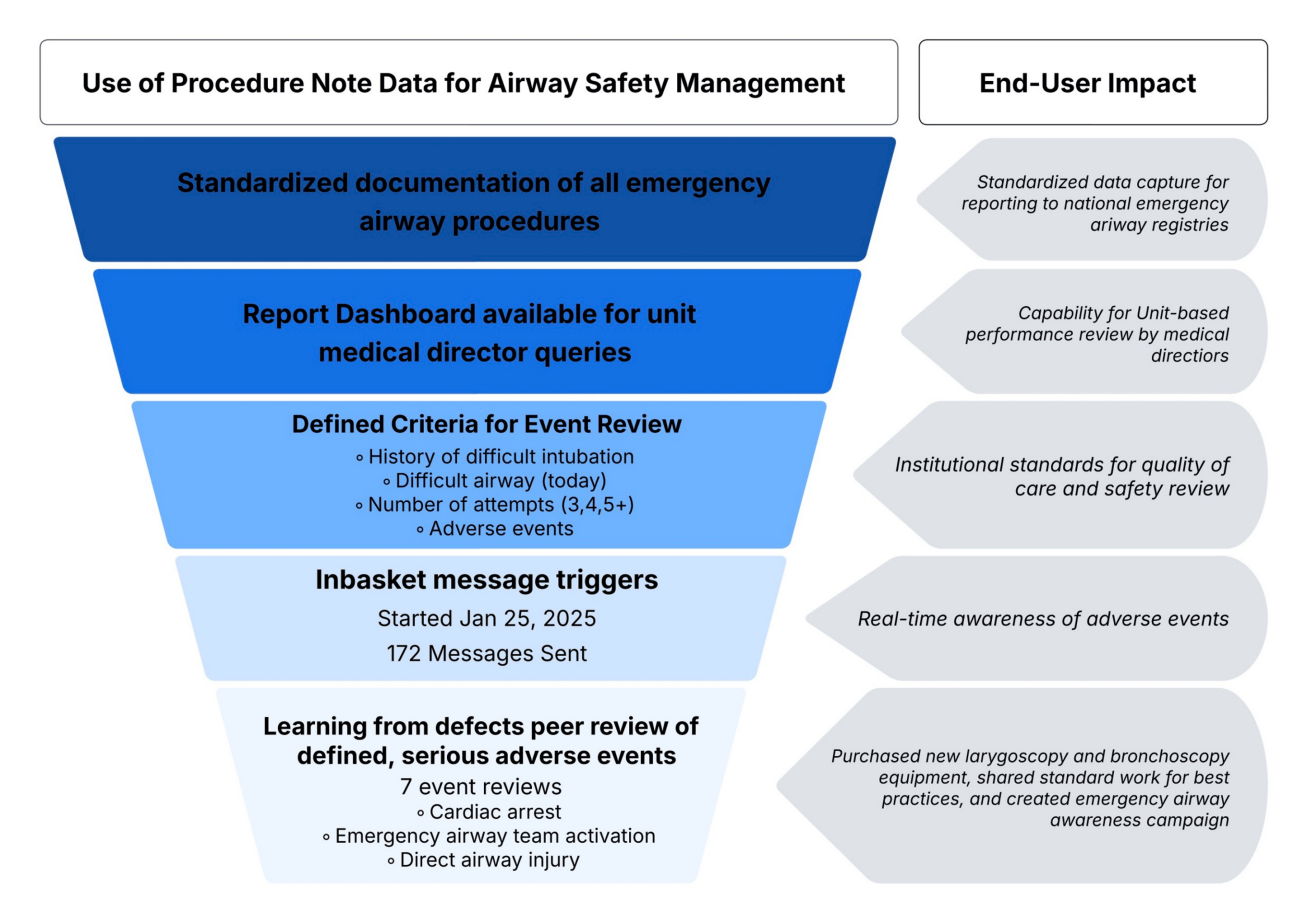
Data-driven emergency airway management workflow Each level in the diagram shows how procedure notes are used for quality and safety work, as well as how they can be filtered to identify key learning cases. This figure is an original work by the authors, created in Lucidchart (www.lucidchart.com).

Secondly, our procedure note has advanced institutional efforts to improve patient outcomes by enabling automated, real-time data sharing with unit medical directors (Figure 3). An EHR-integrated “In Basket” alert is automatically sent to the respective unit medical director when documented airway events meet predefined criteria for review, for example, airway procedures requiring more than three attempts or severe adverse events such as patient death. The In Basket alert provides an opportunity for event review and team support to happen in real time. Standards for event review and peer review of serious events and system-based failures are now possible through an objective and measurable data stream. To date, this push alert system and critical event review have led to the purchase of equipment to fill critical unit-specific equipment gaps, the spread of best-practice safety bundles across units, and a signage campaign with infographics to reinforce critical steps in activating the hospital’s emergency airway response team. 

Lastly, we emphasize that our airway procedure note enables robust, real-time analysis through live EHR-based reporting. These reports can assess outcomes related to airway excellence at the institutional, unit-based, and even individual provider level, such as first-attempt success rates and TI adverse event rates. The granularity of this dataset also allows us to address more complex patient safety questions with ease. For example, we have recently begun tracking first-attempt success for TI and adverse event rates, key quality metrics with strong evidence supporting their impact, yet ones that were not previously documented consistently across both our health system and within each of its unique clinical environments. Further, because the data can be filtered by patient demographics, hospital, unit, provider, and other discrete elements, we are now equipped to generate highly specific insights and develop more precisely targeted interventions.

With regard to the dissemination of the note in the EDs, PICU/PCICU, and NICU, outcomes were in line with our expectations. We expected instant and robust uptake of the note in the EDs given the complete replacement of prior templates with the new one. Because the template was developed and tested with the greatest investment from ED providers, these represented innovators and early adopters [28]. In contrast, we expected other units to require more time to establish new departmental standards, making these early-to-late majority adopters. For the PICU/PCICU, while we expected robust uptake like the ED, we knew additional steps to ensure complete and accurate data capture would be required. The PICU/PCICU had an established process for identifying airway events and manually extracting data for reporting, so we expected that this manual review would continue until we could ensure full uptake and fidelity of the new note. In the NICU, there were various intubation note templates and documentation workflows in use, so we expected that establishing a new, standardized process would take longer to achieve. Furthermore, there is an opportunity to improve fidelity and more comprehensively capture data elements related to airway events.

The high level of uptake in adult ICUs was unexpected. While we collaborated with ICU leaders on making the note available for use in their department, there was no targeted effort to promote its use. Instead, it was through the interest and encouragement of several critical care fellows that the note’s use spread through passive diffusion. While uptake was higher than expected, use of the note remains inconsistent in the adult ICUs, and future steps will require targeted mechanisms to close this gap. 

Our airway procedure note has several strengths, including opportunities for dissemination beyond our health system. We ensured a positive end-user experience through collaboration with documenters who provided feedback on the ease of use and efficiency in our clinical environments. The data collected are aligned with standards set by national repositories, allowing us to contribute to these databases and compare our performance with peer institutions. Local analysis of the data collected has already allowed for the creation of powerful local airway interventions such as automated difficult airway alerts and pre-filled placards. Other benefits of this level of data-gathering include the ability to automatically send immediate messages to division chiefs when a difficult/critical airway procedure has taken place. Additionally, our note allows for documentation of all emergency airway procedures including TI, supraglottic airway device placement, and cricothyrotomy. Finally, the note’s integration into Epic will enable dissemination to other institutions who use Epic. The form, note template, data elements, report columns, and base reports all have Epic building blocks. This means that the build described in this paper can be packaged and delivered to other organizations for them to begin using or adapting.

The direct cost incurred in the development of our airway procedure template is the engagement of informatics staff to create and maintain updates to the note template. Continuous engagement and iterative improvements to our reporting system are driven by this continuous learning framework and will require ongoing informatics support. 

The main limitation of our intervention is that it is dependent on providers to document procedures. It is possible that not all procedures were reported or that they were reported using a different note than ours. Some units performed fidelity checks against their prior established documentation and registry compliance mechanisms, but not all units retrospectively reviewed patient charts in this way, so there is the possibility of data exclusion or inaccuracy. To address this limitation and measure the fidelity of note utilization in the future, we have updated our system configuration so that our procedure notes will now be linked with the nursing and respiratory care airway assessment records in the EHR.

## Conclusions

Standardizing emergency airway procedure documentation using shared electronic data elements is important as it provides a discrete data stream for capturing our current practice, performance outcomes, opportunities, and vulnerabilities. Capitalizing on existing high-reliability workflows and attention to end-user experience during simulation trials contributed to successful dissemination of this template across our health system. This data can be used to drive quality improvement initiatives such as critical event review, difficult airway alerts, and the communication of strategies for difficult airway management across clinical context.

## Figures and Tables

**Figure 4:**
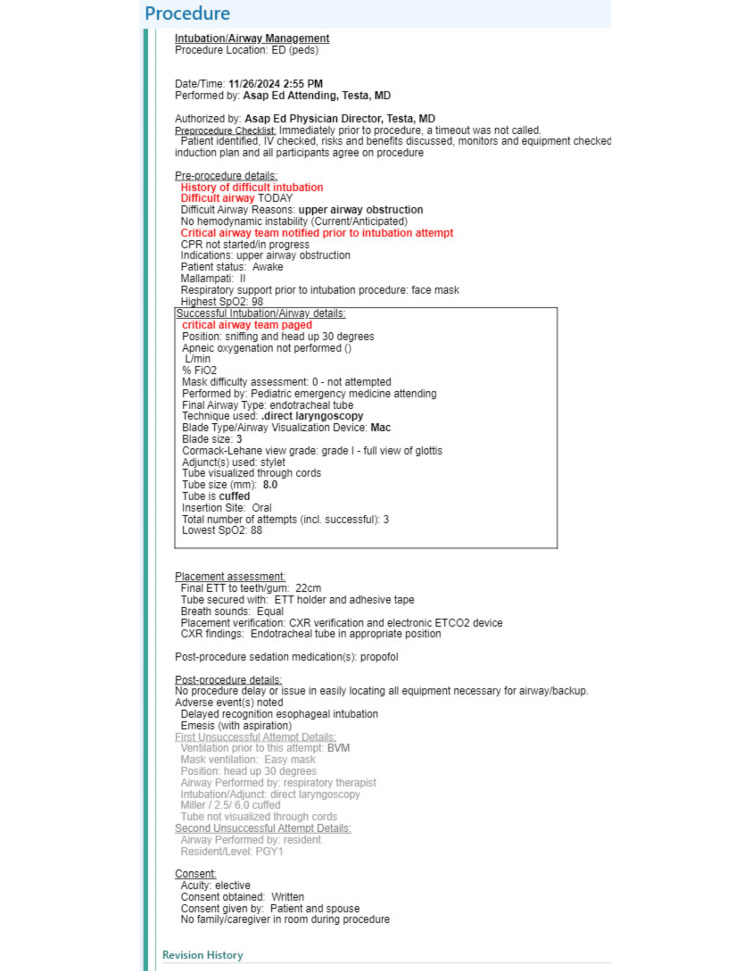
Example of completed airway documentation as it would appear in the electronic health record The boxed section of the note describes details of successful airway management and was designed to draw the attention of patient care teams reviewing prior airway events for a given patient (© 2025 Epic Systems Corporation).
